# AB-DB: Force-Field parameters, MD trajectories, QM-based data, and Descriptors of Antimicrobials

**DOI:** 10.1038/s41597-022-01261-1

**Published:** 2022-04-01

**Authors:** Silvia Gervasoni, Giuliano Malloci, Andrea Bosin, Attilio V. Vargiu, Helen I. Zgurskaya, Paolo Ruggerone

**Affiliations:** 1 University of Cagliari, Department of Physics, I-09042 Monserrato (Cagliari), Italy; 2 University of Oklahoma, Department of Chemistry and Biochemistry, Norman, OK 73072 United States

**Keywords:** Virtual screening, Pharmaceutics, Antimicrobial resistance, Cheminformatics, Electronic structure of atoms and molecules

## Abstract

Antibiotic resistance is a major threat to public health. The development of chemo-informatic tools to guide medicinal chemistry campaigns in the efficint design of antibacterial libraries is urgently needed. We present AB-DB, an open database of all-atom force-field parameters, molecular dynamics trajectories, quantum-mechanical properties, and curated physico-chemical descriptors of antimicrobial compounds. We considered more than 300 molecules belonging to 25 families that include the most relevant antibiotic classes in clinical use, such as *β*-lactams and (fluoro)quinolones, as well as inhibitors of key bacterial proteins. We provide traditional descriptors together with properties obtained with Density Functional Theory calculations. Noteworthy, AB-DB contains less conventional descriptors extracted from *μ*s-long molecular dynamics simulations in explicit solvent. In addition, for each compound we make available force-field parameters for the major micro-species at physiological pH. With the rise of multi-drug-resistant pathogens and the consequent need for novel antibiotics, inhibitors, and drug re-purposing strategies, curated databases containing reliable and not straightforward properties facilitate the integration of data mining and statistics into the discovery of new antimicrobials.

## Abstract (summary)

**Table:** 

Measurement(s)	molecular physical property analysis objective
Technology Type(s)	Computer Modeling

## Background & Summary

The increasing spread of antibiotic resistance in clinics is causing a global health crisis. Mobile genetic element encoding for resistance genes can be transferred among bacterial populations, leading to the need to make more efficient the discovery of new antibiotics and molecules able to improve their efficacy^1,2^. Gram-negative bacteria, such as *Escherichia coli*, *Pseudomonas aeruginosa* and *Acinetobacter baumannii*, are particularly challenging due to the presence of an outer membrane which reduces the permeability of antimicrobials and therefore their efficacy^3–5^. A major obstacle is represented by efflux pumps that act in synergy with the outer membrane ejecting a plethora of compounds with various chemical-physical properties, among which are different classes of antibiotics^6–8^. In addition, inactivating enzymes such as *β*-lactamases^9^ contribute to exacerbate the problem. To date, both academia and industry struggle to identify new antibiotic classes and optimize available compounds^10–15^. Although several strategies have been adopted (e.g., drug repurposing^16^ or systematic exploitation of natural compounds^17^), holistic approaches able to take into account multiple factors contributing to antimicrobial resistance are lacking^18^. Previous works focused primarily on the role of chemical-physical properties of antimicrobial molecules in their accumulation profile, searching for general “rules”^19,20^. For example, O’Shea and Moser^19^ found that antibiotics effective towards Gram-negative bacteria are generally characterized by high molecular weight (MW, around 600 Da) and high polarity (as expressed by cLogD_7.4_ below 0). More recently, Richter *et al*.^21^ identified the presence of a primary amine, flexible bond number (5 or less) and globularity (describing molecular shape), as key features to predict the accumulation of antibiotics in Gram-negative bacteria^22^. Predictive rules of efflux inhibition and avoidance were also identified^23^. This latter study combined standard molecular descriptors to properties derived from structure-based analyses (e.g., interaction descriptors extracted from molecular docking), allowing for a more complete and multi-factorial view. Definition of general rules able to predict both the permeability and the activity of antimicrobial compounds can greatly benefit from the application of machine learning approaches able to speed up the drug discovery process^24,25^. The application of these methods requires collection of data for the learning phase^26,27^, that highlights the need for curated molecular databases providing ready-to-use features.

In this regards, over the years, several molecular databases containing standard descriptors (e.g., PubChem^28^, DrugBank^29^, ChEMBL^30^, ZINC15^31^) or quantum-mechanical (QM) properties^32–36^ have been reported. These data have been extensively used in quantitative-structure-activity-relationships (QSAR) studies^37,38^, and recent works exploited additional information coming from molecular dynamics (MD) simulations^39–43^, that represent an effective tool to address key structural and kinetic features of biological systems^44–47^. However, although freely available servers for the automatic generation of force-field (FF) parameters are available^48–52^, small molecule parameterization remains often a non-trivial task^53^.

**Fig. 1:**
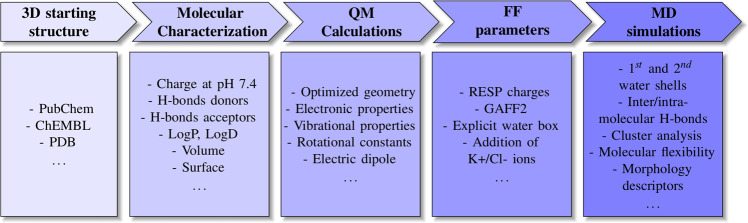
Schematic view of the computational protocol adopted to generate AB-DB. The different steps reporting some representative molecular descriptors are highlighted: molecular characterization, QM calculations, FF parameters generation, MD simulations. For further technical details see Methods section.

**Fig. 2:**
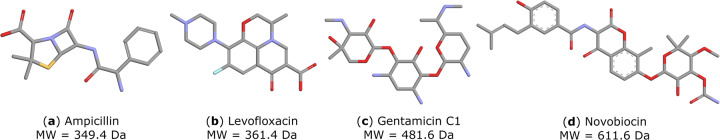
Schematic representation of four representative antimicrobials belonging to different classes, namely (**a**) penicillins, (**b**) (fluoro)quinolones, (**c**) aminoglycosides, and (**d**) aminocoumarins. Graphics rendered with DiscoveryStudio^101^.

Following a previous work^54^, we present a homogeneous database of accurate all-atom FF parameters of more than 300 antimicrobial compounds, together with *μ*s-long MD trajectories and QM-related data (e.g., ground-state optimized geometries). We additionally provide molecular descriptors of different nature: i) classical parameters usually considered in QSAR studies (e.g., MW, atom/ring counts, LogP, …); ii) MD-derived properties (e.g., root-mean-square fluctuations, statistics of intra- and inter-molecular H-bonds, hydration-shells structure and dynamics, …); iii) QM-based parameters (e.g., energies of frontier molecular orbitals, electronic gap, electric dipole moment, …). The computational protocol adopted is schematically depicted in Fig. 1. The molecules considered, ranging in size from cycloserine (13 atoms, MW = 102.09 Da) to rifalazil (132 atoms, MW = 941.09 Da), cover 24 classes of antimicrobial compounds with different mechanisms of action, plus miscellaneous compounds (e.g., fluorescent dyes such as rhodamine 6G and HT33342). In particular, about 30% of compounds in the whole dataset are *β*-lactams, and 10% are inhibitors of key bacterial proteins. High MW compounds (>~1000 Da) such as polymyxin, glycol- and lipo-peptides were omitted from the selection, due to the high computational costs/convergence issues associated to the QM calculation. A schematic depiction of representative compounds showing the overall chemical variability of the sample is given in Fig. 2. The complete set of antimicrobial families and compounds is reported in Table 1.

**Table 1:** List of families and antimicrobial compounds included in AB-DB.

Family	#	Compounds
Aminocoumarins	8	chlorobiocin, novobiocin, declovanillobiocin, isovanillobiocin, novclobiocin 101, plazomicin, **ribostamycin**, vanillobiocin
Aminoglicosides	15	**amikacin**, apramycin, **arbekacin**,** dibekacin**, gentamicin C1, **hygrovetine**, isepamicin, **kanamycin**, **neomycin**, **netilmicin**, **paromomycin**, **sisomicin**, **spectinomycin**, streptomycin, **tobramycin**
Anthracenediones	2	mitoxantrone, pixantrone
Anthracyclines	4	daunorubicin, doxorubicin, epirubicin, idarubicin
*β*-lactamase inhibitors	11	avibactam, bal0029880, clavulanic acid, durlobactam, enmetazobactam, nacubactam, relebactam, sulbactam, tazobactam, thienamycin, zidebactam
Carbapenems	14	biapenem, doripenem, ertapenem, faropenem^1^, imipenem, LK-157, meropenem, olivanic acid, panipenem, razupenem, ritipenem, sanfetrinem, tebipenem, tomopenem
Cephalosporins	39	cefaclor, cefadroxil, cefalonium, cefamandole nafate, cefamandole sodium, cefazolin, cefdinir, cefditoren, cefepime, cefetamet, cefiderocol, cefixime, cefmenoxime, cefmetazole, cefonicid, cefoperazone, ceforanide, cefotaxime, cefotetan, cefoxitin, cefpiramide, cefpirome, cefpodoxime, cefprozil, cefsulodin, ceftaroline, ceftazidime, ceftibuten, ceftizoxime, ceftobiprole, ceftriaxone, cefuroxime, cephalexin, cephaloridine, cephalotin, cephapirin, cephradine, **loracarbef**, nitrocefin
DHFR inhibitors	7	**brodimoprim**, epiroprim, iclaprim (R), iclaprim (S), **tetroxoprim**, **trimethoprim**, triclosan
Efflux pumps inhibitors	9	D13-9001, *MBX2319*, ***MBX2931***, *MBX3132*, *MBX3135*, NMP, PAβN, amitriptyline, chlorpromazine
Quinolones	36	cinoxacin, **ciprofloxacin**, clinafloxacin, danofloxacin, delafloxacin, **difloxacin**, DX-619, **enoxacin**, **enrofloxacin**, **fleroxacin**, flumequine, garenoxacin, gatifloxacin, gemifloxacin, grepafloxacin, **levofloxacin**,** lomefloxacin**, marbofloxacin, moxifloxacin, nadifloxacin, nalidixic acid, **norfloxacin**, **ofloxacin**, orbifloxacin, oxolinic acid, pazufloxacin, **pefloxacin**, pipemidic acid, prulifloxacin, rosoxacin, rufloxacin, **sarafloxacin**, sitafloxacin, sparfloxacin, temafloxacin, trovafloxacin
Fusidanes	2	fusidica acid, helvolic acid
Lincosamides	4	clindamycin, **desalicetin**, lincomycin, pirlimycin
Macrolides	9	azithromycin, *cethromycin*, *clarithromycin*, *dirithromycin*, *erythromycin*, modithromycin, *roxithromycin*, *spiramycin*, *telithromycin*
Monobactams	10	aztreonam, BAL19764, BAL30072, carumonam, gloximonam, nacubactam, nocardicin, oximonam, pirazmonam, tigemonam
Nitrofurans	12	furazolidone, nifurfoline, nifurquinazol, nifurtoinol, nitrofurantoin, nitrovin, nifuratel, nifuroxazide, nifurtimox, nifurzide, nitrofurazone, tinidazole
Nucleosides	4	A-500359A, A-503083E, capuramycin, puromycin
Oxacephem	2	latamoxef, flomoxef
Oxazolidinones	8	contezolid, eperezolid, linezolid, posizolid, radezolid, ranbezolid, sutezolid, tedizolid
Penicillins	20	6-APA, amoxicillin, ampicillin, azlocillin, carbenicillin, cloxacillin, dicloxacillin, epicillin, flucloxacillin, hetacillin, methicillin, mezlocillin, nafcillin, oxacillin, penicillin G, penicillin V, piperacillin, sulbenicillin, temocillin, ticarcillin
Phenicols	5	azidamfenicol, chloramphenicol, florfenicol, tevenel, thiamphenicol
Rifamycins	2	*rifalazil*, ***rifampicin***
Streptogramins	1	dalfopristin
Sulphonamides	20	sulfabenzamide, sulfacetamide, sulfachlorpyridazine, sulfadiazine, sulfadimethoxine, sulfaguanidine, **sulfamerazine**, sulfameter, **sulfamethazine**, sulfamethizole, sulfamethoxazole, sulfamethoxypyridazine, sulfamonomethoxine, sulfamylon,** sulfanitran**, sulfaphenazole, sulfapyridine, sulfaquinoxaline, **sulfathiazole**, sulfisoxazole
Tetracyclines	10	chlortetracycline, demeclocycline, doxycycline, meclocycline, methacycline, minocycline, omadacycline, oxytetracycline, tetracycline, tigecycline
Others	20	acriflavine, cycloserine, dapsone, deoxycholate, *enterobactin*, ethambutol, ethidium, ethionamide, fosfomycin, halicin, HT33342, isoniazid, metronidazole, propidium, pseudomonic acid A, rhodamine 6G, taurocholate, tetraphenylphosphonium, WCK-4234

Boldface labels identify molecules for which two protonation states were considered. Molecules for which the PubChem 3D structure is not available are highlighted in italic. Column “#“ reports the total number of compounds for each family. ^1^Faropenem belongs to penems family, in AB-DB it is included in the carbapenems family.

To the best of our knowledge AB-DB is unique in supplying homogeneously-derived properties of antimicrobial compounds. The accurate FF parameters can be reused for further MD simulations of compounds either alone or interacting with their macromolecular target(s). The MD trajectories can be exploited for ligand- or structure-based studies, in particular for molecular docking. The successful application of this technique requires the knowledge of the bio-active conformation of ligands^55–58^, that is not always found by classical searching algorithms^59,60^. Our curated, homogeneous and not straightforward properties can feed machine learning models towards the discovery of new antimicrobials^61,62^. Input/output files are also supplied to ensure data reproducibility. In the near future we plan to update AB-DB including more compounds, covering additional antimicrobial classes.

## Methods

**Fig. 3:**
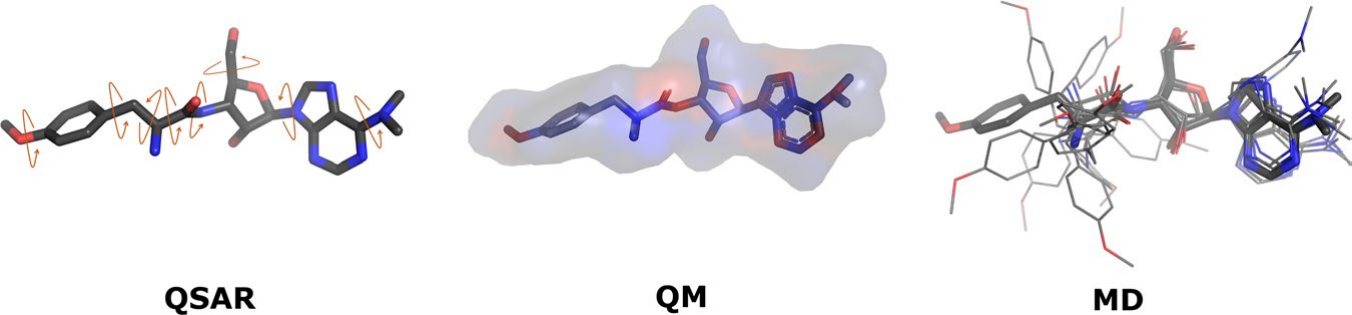
Graphical exemplification of the three types of analysis performed to generate AB-DB. Puromycin molecule has been selected as a test-case. QSAR: number of rotatable bonds (left), QM and FF: atomic partial charges (center), from negative (red) to positive (blue) values, MD: conformations extracted from MD trajectories (right). Graphics rendered with PyMOL^102^.

For each antimicrobial compound we obtained the 3D structure data file (*.sdf* format) from the PubChem database, except for 13 compounds for which the 3D conformation is not available. In those cases, marked in italic in Table 1, the starting structure was taken from the ChEMBL database, or from available X-ray structures. We then used the ChemAxon’s Marvin suite of programs^63^ to calculate the dominant protonation states at physiological pH. For known uncertain cases (e.g., tetracyclines), for which several micro-species with similar population were predicted, the choice has been driven by available experimental data on pK_a_ values. The comparison between the experimental and calculated pK_a_ values of the ionizable groups of a representative set of challenging molecules is reported in Table 2. The large relative errors (even exceeding 100%) were expected for these classes of antimicrobials since pK_a_ determination in Marvin ChemAxon is based on molecular charge distribution, and these molecules are characterized by a complex electronic structure (e.g., with several possible resonance states). For each molecule we then proceeded with QM calculations and FF generation followed by all-atom MD simulations. Properties generated in each step will be referred to as QSAR, MD, and QM descriptors, respectively. The three types of analysis performed in this study are graphically exemplified in Fig. 3 for a test-case molecule and described in details below.

**Table 2:** Average experimental and predicted (boldface) pK_*a*_ values. Relative percentage error of computed pK_*a*_ value is reported in parentheses.

Quinolones					
	pK_*a*_ 1 = carboxylic acid	pK_*a*_ 2 = piperazinyl amine			
ciprofloxacin^103^	6.29, **5.6** (11.7%)	8.26, **8.8** (6.2%)			
difloxacin^103^	5.98,** 5.5** (7.7%)	7.75, **7.0** (9.8%)			
enoxacin^103^	6.57, **5.3** (19.2%)	7.17, **8.7** (21.1%)			
enrofloxacin^103^	6.01, **5.6** (7.7%)	7.93, **7.2** (8.7%)			
fleroxacin^103^	5.78, **5.5** (5.7%)	7.86, **6.5** (17.5%)			
lomefloxacin^103^	5.78, **5.5** (5.7%)	8.74, **8.8** (0.5%)			
norfloxacin^103^	5.97,** 5.6** (6.6%)	8.42, **8.8** (4.2%)			
ofloxacin^103^	6.04, **5.3** (11.5%)	8.09, **6.7** (5.0%)			
pefloxacin^103^	6.45, **5.6** (13.9%)	7.84, **7.0** (10.5%)			
sarafloxacin [104]	5.99, **5.6** (7.4%)	7.84, **8.8** (2.7%)			
levofloxacin^104^	5.6, **5.4** (3.95%)	7.9, **6.7** (15.2%)			
**Sulfonamides**					
	**pK**_***a***_ **1** = **amine**	**pK**_***a***_ **2** = **amide**			
sulfamerazine^103^	2.15, **2.0** (7.0%)	6.82,** 7.0** (2.4%)			
sulfamethazine^103^	2.24, **2.0** (10.7%)	7.51, **7.0** (7.0%)			
sulfathiazole^103^	2.05, **2.0** (0.2%)	7.14, **6.9** (2.9%)			
**Tetracyclines**					
	**pK**_***a***_**1** = **OH(C3)**	**pK**_***a***_**2** =** OH(C12)**	**pK**_***a***_**3 **= **OH(C12)**		
chlortetracycline^103^	3.64, **7.0** (92.6%)	6.57, **8.8** (33.6%)	8.64, **6.18** (28.4%)		
demeclocycline^103^	3.37, **2.6** (22.6%)	7.36,** 8.1** (9.5%)	9.44,** 6.3** (32.9%)		
doxycycline^103^	3.02, **7.3** (142.4%)	7.97, **8.2** (2.3%)	9.15, **5.8** (36.6%)		
meclocycline^103^	4.05, **8.0** (97.3%)	6.87, **5.8** (15.7%)	9.59, **7.0** (26.8%)		
oxytetracycline^103^	3.32, **7.3** (118.4%)	7.02, **8.1** (14.9%)	8.74,** 7.0** (19.7%)		
tetracycline^103^	3.17, **7.2** (127.5%)	6.79, **7.8** (29.9%)	9.07, **6.2** (31.4%)		
**Aminoglycosides**					
	**pK**_***a***_**1** = **N1**	**pK**_***a***_**2** = **N3**	**pK**_***a***_**3** = **N2’**	**pK**_***a***_**4 **=** N6’**	**pK**_***a***_**5 **=** N3”**
amikacin^105^	9.89,** 9.6** (2.8%)	7.64, **9.0** (17.5%)	—	8.81, **8.2** (6.9%)	8.05, **8.4** (4.6%)
gentamicin C1^106^	7.67, **9.5** (23.9%)	6.19, **9.0** (45.4%)	7.4, **10.1** (36.5%)	9.86,** 8.5** (13.8%)	8.78, **7.41** (15.6%)
netilmicin^105^	8.15,** 9.2** (12.9%)	6.52, **8.3** (27.3%)	8.15, **7.2** (11.7%)	9.32,** 8.8** (5.6%)	8.48, **9.7** (14.4%)
sisomicin^105^	7.43,** 9.2** (23.8%)	6.21, **8.3** (33.7%)	8.01, **7.4** (7.6%)	9.31, **8.8** (5.5%)	8.50, **9.6** (11.5%)
tobramycin^105^	7.56, **9.5** (25.7%)	6.70, **7.4** (9.9%)	7.75, **8.1** (5.0%)	9.11, **9.0** (1.0%)	7.71, **8.6** (11.7%)
**Cephalosporins**					
	**pK**_***a***_ = **Amine**				
loracarbef^107^	6.84, **7.2** (5.4%)				
**DHFR inhibitors**					
	**pK**_***a***_ = **diaminopyrimidine**				
trimethoprim^108^	7.50, **7.2** (4.5%)				
**Rifamycins**					
	**pK**_***a***_**1**= **4-hydroxy**	**pK**_***a***_**2**= **3-piperazine nitrogen**			
rifampicin^109^	1.7, **10.0** (485.9%)	7.9, **7.1** (9.8%)

### Quantum-mechanical calculations and force-field generation

The 3D structures of the molecules downloaded from the PubChem or ChEMBL databases (see above) underwent quantum-chemical calculations at the Density Functional Theory (DFT) level^64^, using the Gaussian16 package^65^. We employed the hybrid B3LYP functional^66^, in conjunction with the split-valence 6–31G** Gaussian basis-set^67^. The combination B3LYP/6–31G** represents a good compromise between accuracy and computational cost and is widely used for small molecules^68–70^. In all cases we disabled molecular symmetry (Symmetry = None), adopted restrictive convergence criteria for self-consistent-field iterations (10^−8^ Ha, SCF(Conver = 8)), and used a pruned (99,590) grid (Int = UltraFine) for numerical integration. For the few cases for which convergence criteria were not reached, geometry optimization was first performed with a smaller basis-set (6–31G) and converged geometry and molecular orbitals were used as a starting point for the subsequent B3LYP/6–31G** step. For each compound we optimized the ground-state structure employing the Polarizable Continuum Model^71^ to mimic the effect of water solvent (SCRF = (PCM,Solvent = Water)), particularly to avoid formation of strong intra-molecular H-bonds. We then performed full vibrational analyses obtaining real frequencies in all cases, thus confirming the geometries obtained to be global minima. We processed the output of Gaussian16 with GaussSum^72^ to extract molecular orbital data. On the optimized geometry we then performed B3LYP/6–31G** single-point energy calculations in vacuum to generate the atomic partial charges fitting the molecular electrostatic potential. We used the Merz-Kollman scheme^73^ to construct a grid of points around the molecule under the constraint of reproducing the overall electric dipole moment of the molecule (Pop = (ESP,Dipole,Regular)). The two-step restrained electrostatic potential (RESP) method^74^ implemented in the Antechamber package^75^ was used to generate atomic partial charges at the DFT level, instead of the automatic AM1-BCC charges^76^. This step enabled the generation of the FF files using the General Amber Force Field 2 (GAFF2)^77^. In a single case, namely the siderophore enterobactin loaded with Fe(III)^78^, FF files were obtained using the metal center parameter builder module^79^ of the Amber18 package^80^, slightly modified accordingly to the QM settings described above.

### Molecular dynamics simulations

All-atom MD simulations were performed in explicit water solution using Amber18. Systems were solvated within a box of TIP3P water model^81^ and K^+^/Cl^−^ counter ions^82^, to reach an ionic concentration of 0.1 M, using the program tleap of Amber18^80^. GAFF2 parameters obtained as described above were adopted for antimicrobial compounds. All systems underwent an energy minimization, a heating followed by a cooling phase, and a short productive dynamics to relax the simulation box. Finally the production 1 *μ*s-long MD simulation was performed, under the NPT ensemble (1 Atm and 310 K) using the isotropic Berendsen barostat^83^ and the Langevin thermostat^84^. Further details on MD settings can be found in ref. ^54^.

### Descriptors generation

From the output of QM and MD simulations we extracted all molecular descriptors (~80 in total for each compound, see list in Table S1). Most QSAR descriptors were computed on the QM optimzed geometries using the calculator plugin of the Marvin ChemAxon program^63^. Given the importance of octanol/water partition coefficient in drug design^85^, we provide an additional estimate of this parameter by means of the XLOGP3 program^86^. Furthermore, for each compound we derived the molecular properties associated with the “entry rules”, a series of guidelines that have been recently proposed to increase small-molecule accumulation in Gram-negative bacteria^21^. QM-based properties were obtained from the Gaussian16 output files of the implicit-solvent geometry optimization. Isotropic and anisotropic polarizabilities were derived from the polarizability tensor according to ref. ^87^. We additionally provided the molecular dipole moment in vacuum consistent with the atomic partial charges of the FF files, computed as described above. From the all-atom MD simulations we obtained structural and dynamical features by means of the CPPTRAJ program^88^. First and second water shells were extracted using a lower (upper) cutoff of 3.4 (5.0) Å. For the analysis of intra- and inter-molecular H-bonds we adopted angle and distance cutoffs of 135° (donor-hydrogen-acceptor angle) and 3.5 Å (donor-acceptor), respectively^89^. The number and population of structural clusters were determined using a hierarchical agglomerative algorithm^90^ and the molecule root-mean-squaredeviation (RMSD) value as a metric. To evaluate atomic root-mean-square fluctuations (RMSF) we used the utility g_rmsf of the GROMACS package^91^. During the MD runs we also monitored three morphology descriptors related to the gyration tensor, i.e., asphericity, acylindricity, and kappa2, as implemented in the PLUMED plugin^92^. Asphericity and acylindricity give a measure of the deviation of the mass distribution from spherical and cylindrical symmetry, respectively; the relative shape anisotropy kappa2 is limited between 0 and 1 and reflects both symmetry and dimensionality^93^. The minimal projection area (MPA) is the minimum of the circular areas projected perpendicularly to the principal axes of inertia of the molecule, calculated based on the atomic van der Waals radii (Å). The dynamical evolution of the MPA have been determined with the combined use of Open Babel^94^ and ChemAxon’s calculator plugin^63^.

## Data Records

**Fig. 4:**
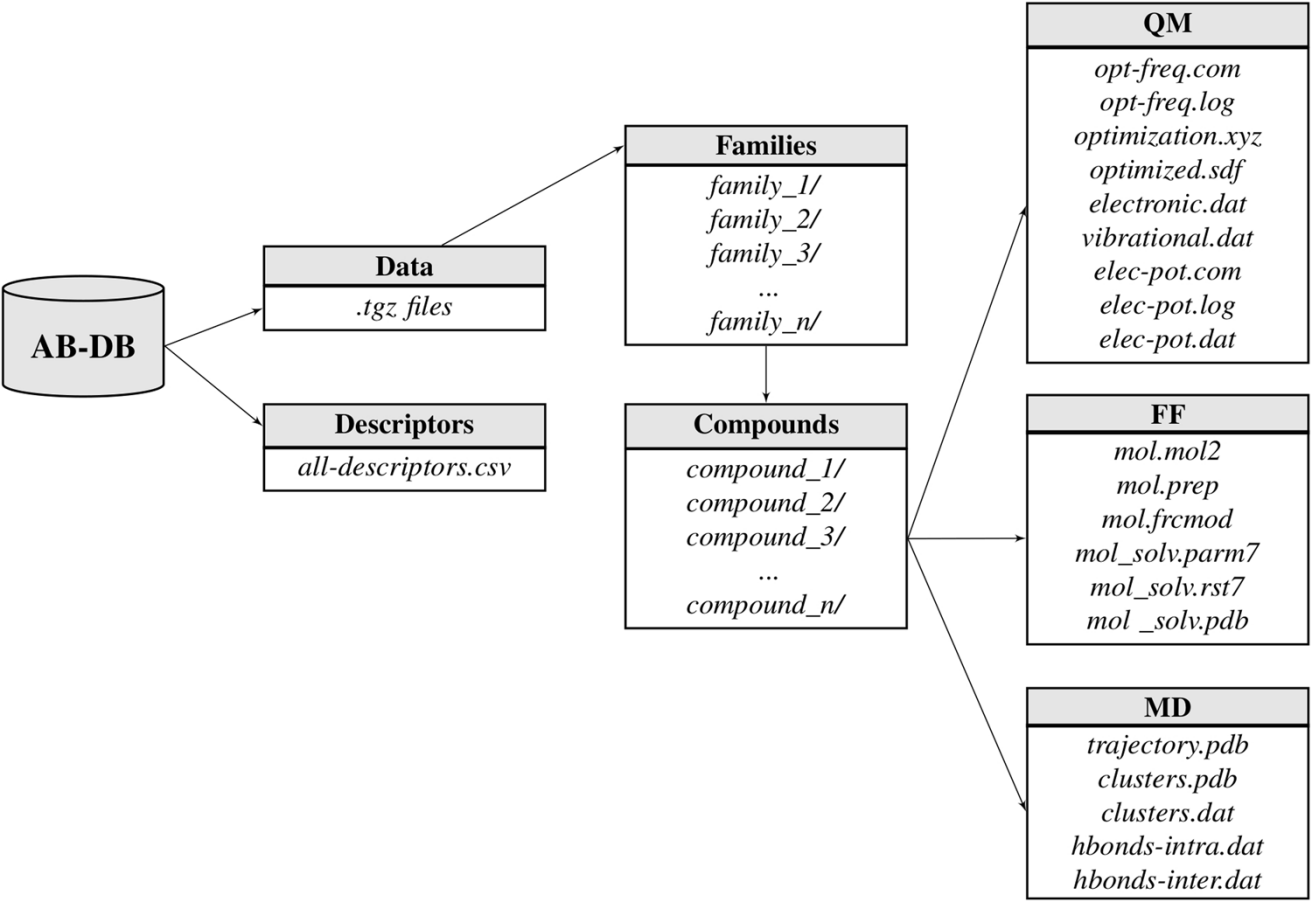
Schematic representation of the AB-DB structure reporting filenames of each sub-directory.

AB-DB is available on figshare^95^. The computed molecular descriptors are given in the comma separated file *all-descriptors.csv*. A compressed TAR archive for each family is provided (e.g., *carbapenems.tgz*). In turn, every archive contains sub-folders named after the compound and the net charge considered in the calculations (e.g., *carbapenems/ertapenem_-1/*). For 34 molecules the two protonation states most populated at pH = 7.4 were considered (see Methods section). In these cases two folders per compound are reported, with different values of the net charge (e.g., *quinolones/ciprofloxacin_0/* where the compound is considered as zwitterionic, and *quinolones/ciprofloxacin_-1/* where the nitrogen atom of the piperazine ring is considered in its neutral form). Each compound folder contains a 2D sketch of the molecule (*2d.png*), and a total of 20 files distributed into three sub-directories reporting QM (*QM/*), FF (*FF/*), and MD (*MD/*) data. Figure 4 shows a schematic representation of the database structure describing the path of all files provided.

### Quantum-mechanical data

*QM/* folders contain files derived from QM calculations (see Quantum-mechanical calculations and force-field generation section). In details, the *opt-freq.com* and *opt-freq.log* are the input and output files of the Gaussian16 geometry optimization and frequency analysis in implicit solvent. The minimization steps are collected in the *optimization.xyz* file and the final optimized structure is given in structure data file format as *optimized.sdf*. This file, generated with Open Babel^94^ from the corresponding .xyz file and carefully checked manually, is also provided for reproducibility purposes since it has been used to compute QSAR descriptors. We also collected the electronic structure and the harmonic vibrational frequencies into *electronic.dat* and *vibrational.dat* files, respectively. The *elec-pot.com* and *elec-pot.log* are respectively the input and output files of the Gaussian16 single-point energy calculation in vacuum, performed to derive atomic partial charges. The resulting electrostatic potential file is *elec-pot.dat*.

### Force-field data

For each compound we supply in the corresponding *FF/* folder the *mol.mol2* and Amber *mol.prep* files, containing the optimized structure of the molecule with RESP partial charges. The Amber force-field modification file *mol.frcmod* with all parameters not included in the GAFF2 is also provided. For reproducibility purposes we make available the Amber parameter/topology *mol_solv.parm7*, and coordinate/restart *mol_solv.rst7* files used to perform the MD simulation in explicit solvent. The corresponding *mol_solv.pdb* file generated using the ambpdb program^80^ is also provided.

### Molecular dynamics data

*MD/* folders store the *μ*s-long MD trajectories performed in explicit water solution (see Molecular dynamics simulations section) in the file *trajectory.pdb* (100000 frames). The representatives of the ten most populated clusters extracted from the trajectory are given in *clusters.pdb*, and their corresponding fraction in *clusters.dat*. The statistics of intra- and inter-molecular H-bonds are collected in *hbonds-intra.dat* and *hbonds-inter.dat*, respectively.

## Technical Validation

AB-DB is built making use of the different computational steps detailed above: molecular characterization, QM calculations, FF generation, MD simulations, and extraction of physico-chemical descriptors. Concerning the starting configurations used for the subsequent steps, we carefully checked the protonation state of all compounds at physiological pH, paying particular attention to uncertain cases with two major populated species (see Table 2 and Methods section for details). In details, for these ambiguous cases, we searched the literature for experimental values of pK_a_, that were consistently used as reference throughout the class. As for the QM calculations, the DFT level of theory adopted is routinely used and has proven to be reliable for small organic molecules, providing FF parameters compatible with available FFs for macromolecules^50^. B3LYP/6–31G** calculations represent a good compromise between accuracy and computational cost^96^. Therefore, no further validation is here provided for QM calculations. In the following we present a thorough justification of the reliability of our data through the comparison with experiments.

### Descriptors validation

Calculation of classical parameters reporting the topological properties of molecules, such as the number of atoms or the count of aliphatic bonds, is quite straightforward. Most popular databases (e.g. PubChem, DrugBank) indeed employ ChemAxon’s tools to automatically compute these properties. In AB-DB, we likewise used the same programs to obtain the QSAR descriptors. However, for LogP, which is known to be a key feature for antimicrobial penetration kinetics^61,97,98^, we exploited another widely used method (XLOGP3, see Methods section). Note that accurate prediction of LogP is a well-known challenge in computational chemistry, and is also common to find severe disagreement through experimental results obtained for the same compound^99,100^. In order to assess the quality of our predictions, we collected available experimental LogP values for a subset of molecules. Table 3 compares the experimental data, falling in the range [−1.69, 5.15], with the computed ones, highlighting the differences between the two methods. In most cases the two predicted values are similar and agree with the experimental LogP. However, as expected, ambiguous situations were also found. Methicillin, for instance, was well predicted by XLOGP3 (computed 1.96 vs. experimental ~1.90) while cxcalc yielded a poor estimation (computed 0.79). On the contrary, the latter program agrees with experiments for lomefloxacin (computed −0.43 vs. experimental −0.47), whereas the former failed (computed 0.27).

**Table 3:** Experimental logP (LogP exp) and LogP values computed by XLOGP3 and cxcalc.

Family	Compound	LogP exp	XLOGP3 (%ERR)	LogP cxcalc (%ERR)
aminocoumarins	chlorobiocin	5.15^110^	5.98 (16)	4.94 (4)
	novobiocin	3.1^111^	3.96 (28)	3.26 (5)
streptogramins	dalfopristin	2.57^99^	2.23 (13)	1.58 (39)
macrolides	telithromycin	2.1^112^	4.16 (98)	5.05 (140)
penicillins	dicloxacill	2.91^113^	3.78 (30)	2.91 (0)
	cloxacillin	2.43^113^	3.15 (30)	2.30 (5)
	oxacillin	2.31^113^	2.53 (10)	1.70 (26)
	penicillin G	1.70^113^	1.95 (15)	1.08 (36)
	methicillin	1.896^114^	1.96 (3)	0.79 (58)
	temocillin	1.39^99^	2.4 (73)	1.2 (14)
quinolones	delafloxacin	2.63^99^	2.76 (5)	2.56 (3)
	difloxacin	0.84^100^	1.49 (77)	1.75 (108)
	grepafloxacin	0.66^115^	0.72 (9)	0.07 (89)
	sitafloxacin	−0.16^116^	0.76 (575)	−0.17 (6)
	lomefloxacin	−0.47^117^	0.27 (157)	−0.43 (9)
cephalosporins	cefpiramide	0.95^99^	0.84 (12)	−0.97 (202)
	cefotetan	0.31^99^	0.64 (106)	−0.38 (223)
	ceforanide	−1.35^99^	−1.87 (39)	−3.18 (136)
lincosamides	clindamycin	0.78^118^	1.76 (126)	1.04 (33)
*β*-lactamase inhibitors	tazobactam	−1.69^99^	−1.33 (21)	−1.4 (17)

Relative error (%) is reported in parentheses.

### Validation of force-field parameters and molecular dynamics trajectories

To assess the reliability of the FF generated for all compounds we computed the RMSD between the QM B3LYP/6–31G** optimized geometry and the molecular mechanics minimum-energy structure obtained with the GAFF2 parameters of the database. Table 4 shows the good agreement between the two sets of structures, differing on average by less than 1 Å, with an overall mean value of 0.5 ± 0.1 Å. The registered low RMSDs prove the accuracy of the FF parameters presented in AB-DB and used for the MD simulations.

**Table 4:** Average RMSDs and standard deviations (Å) between the DFT and molecular mechanics optimized geometries for the classes of antimicrobial compounds included in AB-DB.

Family	Mean ± STD
aminocoumarins	0.4 ± 0.1
aminoglycosides	0.3 ± 0.1
anthracyclines	0.4 ± 0.1
beta-lactamase-inhibitors	0.3 ± 0.2
carbapenems	0.5 ± 0.2
cephalosporins	0.6 ± 0.2
dhfr-inhibitors	0.4 ± 0.2
efflux-pumps-inhibitors	0.4 ± 0.2
fusidanes	0.4 ± 0.1
lincosamides	0.4 ± 0.2
macrolides	0.4 ± 0.1
monobactams	0.6 ± 0.1
nucleosides	0.6 ± 0.2
oxacephem	0.5 ± 0.1
oxazolidinones	0.4 ± 0.1
penicillins	0.5 ± 0.2
phenicols	0.5 ± 0.1
quinolones	0.3 ± 0.1
rifamycins	0.5 ± 0.1
streptogramins	0.6 ± 0.0
sulphonamides	0.6 ± 0.3
tetracyclines	0.4 ± 0.3
others	0.3 ± 0.2

To give a measure of the quality of MD simulations, we compared the representative conformations extracted from MD trajectories (cluster representatives) of selected compounds with their 3D experimental structure available on the Protein Data Bank, in complex with biological targets. When multiple experimental structures were available for the same compound, we considered the one with the highest resolution. The total number of 85 experimental structures collected are listed in Table S2, reporting the corresponding PDB code and the weighted average RMSDs (<RMDS>_*w*_), obtained using cluster populations as weights. The average values associated to selected families are also given. The mean value of <RMDS>_*w*_ considering all families is 1.8 ± 0.8 Å, with the highest and smallest value reached by aminocoumarins (3.6 ± 0.4 Å) and tetracyclines (0.9 ± 0.2 Å), respectively. As expected, bigger and more flexible molecules give rise to higher <RMDS>_*w*_, whereas smaller and more rigid compounds show lower values. Overall, the performed MD simulations based on GAFF2 parameters appear to be able to sample molecular conformations found in available experimental structures.

As emphasized in previous works^54,60^, MD simulations enable to go beyond a static picture of molecules, providing ranges of properties accounting for their dynamical nature and their impact on biological activity. Prominent examples are represented by MD simulations performed to differentiate the most active inhibitors of ERK2 kinase^39^ and Ptch1 multidrug efflux transporter^58^.

## Supplementary information

Table S1

Table S2

## Data Availability

QSAR calculations were performed using the ChemAxon’s Marvin suite of programs, version 21.14^63^. For QM calculations we used the Gaussian16 package, revision A.03^65^. The Amber18 package^80^ was used for MD simulations and FF generation. We used simple bash scripts to iteratively extract descriptors from outputs and generate AB-DB data-files.
