# Supplementary material for: AB-DB: Force-Field parameters, MD trajectories, QM-based data, and Descriptors of Antimicrobials
Source: Sci Data. 2022 Apr 1;9:148. doi: 10.1038/s41597-022-01261-1 (PMC8976083; doi:10.1038/s41597-022-01261-1)
Supplement: Supplementary file 1 — Table S1 [file 41597_2022_1261_MOESM1_ESM.pdf]

QSAR descriptors (51)

Name	Data type (Unit)	Description
Formula	S	Molecular formula
Smiles	S	SMILES string
MW	F (Da)	Molecular weight
Charge	I (e)	Molecular charge
Atoms	I	Number of atoms
Heavy_atoms	I	Number of non-Hydrogen atoms
Asymmetric_atoms	I	Number of chiral atoms
Aliphatic_atoms	I	Number of aliphatic atoms
Aromatic_atoms	I	Number of aromatic atoms
Bonds	I	Number of bonds
Aliphatic_bonds	I	Number of liphatic bonds
Aromatic_bonds	I	Number of aromatic bonds
Rings	I	Number of rings
Aliphatic_rings	I	Number of aliphatic rings
Aromatic_rings	I	Number of aromatic rings
Heterorings	I	Number of heterorings
Chainatoms	I	Number of chain atoms
Chainbonds	I	Number of chain bonds
Refractivity	F	Refractivity
Balabanindex	F	Balaban index
Hararyindex	F	Harary index
Plattindex	I	Platt index
Randicindex	F	Randic index
Szegedindex	I	Szeged index
Wienerindex	I	Wiener index
Hyperwienerindex	I	Hyperwiener index
Donors	I	Number of Hydrogen bond donors
Acceptors	I	Number of Hydrogen bond acceptors
Surface	F (Å^2)	Molecular surface area (3D)
Topological_surface	F (Å^2)	Topological surface area (2D)
ASA	F (Å^2)	Water accessible surface area
ASAplus	F (Å^2)	Positive accessible surface area
ASAminus	F (Å^2)	Negative accessible surface area
ASA_H	F (Å^2)	Hydrophobic surface area
ASA_P	F (Å^2)	Polar surface area
ASAplus/ASA	F	Ratio ASAplus/ASA
ASAminus/ASA	F	Ratio ASAminus/ASA
ASA_H/ASA	F	Ratio ASA_H/ASA
ASA_P/ASA	F	Ratio ASA_P/ASA
Pienergy	F	Pi energy
FSP3	F	Fraction of sp^3 carbons
Cyclomatic_number	I	Cyclomatic number
Volume	F (Å^3)	Van der waals volume
logP	F	Octanol-water partition coefficient logP
logD	F	logD at pH=7.4
XLOGP3	F	Octanol-water partition coefficient as computed with the XLOGP3 software[?]
GLOB	F	Globularity of the molecule[?]
PBF	F	Average distance to the plane of best fit[?]
RB	I	Number of rotatable bonds[?]
Primary_amine	B	Presence of primary amine[?]
ZWITT	B	Zwitterionic species

QM descriptors (13)

DFT_ENE	F (a.u.)	Total DFT energy from B3LYP-6-31G** optimization in implicit solvent
HOMO	F (eV)	Energy associated to the highest occupied molecular orbital (HOMO)
LUMO	F (eV)	Energy associated to the lowest unoccupied molecular orbital (LUMO)
GAP	F (eV)	HOMO-LUMO energy gap
DIP	F (Db)	Total dipole moment of the molecule extracted from single-point energy calculation in vacuum (at the optimized geometry in implicit solvent). This value is consistent with the atomic partial charges adopted for MD simulations in explicit water solution
POL_ISO	F ($Å^3$)	Isotropic dipole polarizability computed from polarizability tensor
POL_ANI	F ($Å^3$)	Anisotropic dipole polarizability computed from polarizability tensor
ROT_A	F (GHz)	Rotational constant A of the molecular configuration optimized in implicit solvent
ROT_B	F (GHz)	Rotational constant B of the molecular configuration optimized in implicit solvent
ROT_C	F (GHz)	Rotational constant C of the molecular configuration optimized in implicit solvent
E_TH	F (kcal/mol)	Internal thermal energy from thermochemical analysis
CV	F (cal/mol-K)	Constant-volume heat capacity from thermochemical analysis
S	F (cal/mol-K)	Entropy from thermochemical analysis

MD descriptors (14)

WAT1 $\pm$ ERR_WAT1	I	Average number and standard deviation of water molecules in the 1st solvation shell
WAT2 $\pm$ ERR_WAT2	I	Average number and standard deviation of water molecules in the 2nd solvation shell
RMSF $\pm$ ERR_RMSF	F (Å)	Average value and standard deviation of the RMSF of atomic positions in MD trajectory
MIN-PA $\pm$ ERR_MIN-PA	F ($Å^2$)	Average value and standard deviation of the minimal projection area associated to the configurations explored by the molecule during MD trajectory
ASP $\pm$ ERR_ASP	F	Average asphericity and standard deviation computed along MD. This parameter gives a measure of the deviation of the mass distribution from spherical symmetry
ACY $\pm$ ERR_ACY	F	Average acylindricity and standard deviation computed along MD. This parameter gives a measure of the deviation of the mass distribution from cylindrical symmetry
K2 $\pm$ ERR_K2	F	Average relative shape anisotropy kappa2 and standard deviation computed along MD. This parameter reflects both symmetry and dimensionality of the molecule

Table S1. List of molecular descriptors provided for each compound (file *all-descriptors.csv*). The total number of descriptors of each type (QSAR, QM, MD) is given between parentheses. Possible data types are string (S), integer (I), float (F), and boolean (B). QSAR descriptors were computed using the Marvin ChemAxon calculator plugin cxcalc, with the exception of XLOGP3 and the eNTRy Rules parameters GLOB, PBF, RB, and Primary_amine.
