# Supplementary material for: AB-DB: Force-Field parameters, MD trajectories, QM-based data, and Descriptors of Antimicrobials
Source: Sci Data. 2022 Apr 1;9:148. doi: 10.1038/s41597-022-01261-1 (PMC8976083; doi:10.1038/s41597-022-01261-1)
Supplement: Supplementary file 2 — Table S2 [file 41597_2022_1261_MOESM2_ESM.pdf]

Family	Compounds	PDB Id (Res)	$<RMDS>_w$	$\overline{<RMDS>_w} \pm$ Std
aminocoumarins	chlorobiocin	1KZN (2.3)	4.2	3.9 ± 0.4
	novobiocin	6Y8L (1.4)	3.6	
aminoglycosides	amikacin	6VTA (1.42)	2.1	2.2 ± 0.8
	arbekacin	6CGG (2.4)	3.0	
	dibekacin	6CAV (2.6)	3.3	
	gentamicin	5CFT (1.5)	2.4	
	kanamycin	6CTZ (1.34)	1.5	
	neomycin	6UN8 (1.65)	1.8	
	paromomycin	2VQY (1.8)	1.8	
	sisomicin	6NP2 (1.2)	2.5	
	spectinomycin	6XZ0 (2.8)	0.6	
	streptomycin	6FUX (2.05)	1.7	
	tobramycin	6NP4 (1.15)	3.3	
anthracyclines	daunorubicin	3TVB (1.08)	1.2	1.2 ± 0.1
	doxorubicin	1P20 (1.34)	1.3	
b-lactamase inhibitors	avibactam	7KEP (2.32)	1.1	1.0 ± 0.2
	clavulanic acid	2XF3 (1.55)	0.7	
	sulbatctam	4FH2 (1.44)	1.2	
	tazobactam	1VM1 (2.02)	0.9	
carbapenems	doripenem	6P9C (1.9)	1.5	1.2 ± 0.3
	meropenem	4EUZ (1.08)	1.2	
	faropenem	3BFF (1.9)	0.9	
cephalosporins	cefamandole sodium	3NY4 (1.22)	2.4	2.1 ± 0.4
	cefepime	5OJ0 (3.06)	2.0	
	cefixime	4KOU (1.6)	2.3	
	cefmetazole	4KOS (1.55)	1.9	
	cefotaxime	6C79 (1.1)	1.7	
	cefoxitin	4KOW (1.45)	1.5	
	ceftriaxone	5NZX (1.47)	2.6	
	cefuroxime	4KOV (1.6)	2.4	
	cephalotin	1KVL (1.53)	1.7	
DHFR-inhibitors	iclaprim(R)	3FYW (2.1)	1.5	1.4 ± 0.2
	trimethoprim	4KM2 (1.4)	1.5	
	triclosan	6AH9 (1.74)	1.1	
efflux pumps inhibitors	amitriptyline	3APV (2.15)	1.3	2.8 ± 1.1
	chlorpromazine	5G08 (1.52)	1.1	
	D13-9001	3W9H (3.05)	3.0	
	MBX2319	5ENO (2.2)	2.7	
	MBX2931	5ENP (1.9)	3.5	
	MBX3132	5ENQ (1.8)	3.7	
	MBX3135	5ENR (2.3)	4.0	
fusidanes	fusidic acid	7A1U (1.67)	1.1	1.1
lincosamides	clindamycin	3JZ0 (2)	2.5	2.5
	lincomycin	4WH5 (1.82)	2.5	
macrolides	azithromycin	5IGI (1.2)	1.0	2.4 ± 1.5
	cethromycin	1NWX (3.5)	1.5	
	clarithromycin	5IGJ (1.4)	1.8	
	dirithromycin	6XZ7 (2.1)	4.2	
	erythromycin	5IWU (1.3)	1.8	
	roxithromycin	1JZZ (3.8)	2.8	
	spiromycin	5IGZ (1.6)	1.2	
	telithromycin	1P9X (3.4)	5.2	
nucleosides	puromycin	5YO1 (2.5)	1.8	1.8
oxazolidinones	linezolid	5NZ0 (1.825)	1.6	1.5
	sutezolid	5NZ1 (2.33)	1.5	
penicillins	ampicillin	3NDV (1.7)	1.6	2.2 ± 0.6
	nafcillin	1T9W (3.23)	2.8	
	penicillin G	5KMW (1.1)	1.6	

	penicillin V	2Z71 (2.6)	2.1	
	piperacillin	3Q07 (1.5)	2.7	
phenicols	chloramphenicol	4OAE (1.25)	1.6	1.6
quinolones	ciprofloxacin	4BVV (1.8)	0.9	1.0 ± 0.2
	clinafloxacin	3RAD (3.35)	1.0	
	gatifloxacin	5BTD (2.5)	0.9	
	levofloxacin	5BTG (2.5)	0.7	
	moxifloxacin	5BS8 (2.399)	1.2	
	trovafloxacin	4KOE (3.02)	1.4	
rifamycins	rifampicin	2HW2 (1.45)	2.9	2.9
sulphonamides	sulfabenzamide	3RCF (1.15)	1.6	1.4 ± 0.2
	sulfamethoxazole	6YSX (1.48)	1.4	
	sulfapyridine	5RF8 (1.44)	1.5	
	sulfathiazole	5G44 (2.24)	1.2	
tetracyclines	chlortetracycline	5TUI (2.15)	0.6	0.8 ± 0.2
	doxycycline	5OM2 (1.47)	0.6	
	minocycline	2XPV (1.49)	1.0	
	oxytetracycline	2XPW (1.44)	0.7	
	tetracycline	6FPL (1.027)	0.9	
	tigecycline	4A6N (2.3)	1.1	
others	ethidium	5ER5 (1.26)	0.4	1.4 ± 0.8
	fosfomycin	1LQP (1.9)	0.8	
	propidium	1N5R (2.25)	1.4	
	pseudomonic acid A	1FFY (2.2)	2.9	
	rhodamine 6G	5KAW (1.86)	1.2	
	taurocholate	6PXA (1.82)	2.0	
	tetraphenylphosponium	5KAW (1.86)	1.4	

Table S2. Mean RMSD between the cluster conformations resulting from the MD simulation in explicit solvent and the highest resolution crystal structure available for the molecule. The average RMSD values (Å) are weighted on cluster population (<RMDS>$_w$), and the mean value of <RMDS>$_w$ for each family with the corresponding standard deviation is also reported ($\overline{< RMDS >}_w \pm$ Std). The PDB Id and the resolution (Å) of the considered experimental structure is also listed.
